# ANCA-associated vasculitis with crescentic glomerulonephritis and papillary thyroid carcinoma: A case report

**DOI:** 10.1097/MD.0000000000044839

**Published:** 2025-09-26

**Authors:** Juan Chen, Linna Lu, Kun He, Diansheng Huang, Zhandong Meng, Yuanfa Li, Xin Xiang

**Affiliations:** a The Department of Rheumatology, Minzu Hospital of Guangxi Zhuang Autonomous Region, Nanning, China.

**Keywords:** anticytoplasmic antibody (ANCA), cancer, case report, crescentic glomerulonephritis, papillary thyroid carcinoma

## Abstract

**Rationale:**

ANCA-associated vasculitis (AAV) is an autoimmune disease. Renal involvement in AAV can manifest as crescentic glomerulonephritis (CGN), which can lead to rapidly progressive renal failure. AAV with crescentic glomerulonephritis complicated and papillary thyroid carcinoma remains rare. The purpose of this report was to enhance the understanding of the diagnosis and treatment of AAV combined with crescentic glomerulonephritis and papillary thyroid carcinoma, reduce misdiagnosis and missed diagnosis, and for the patient with AAV, attention should be paid to screening for cancer, and multi-disciplinary collaboration is required for diagnosis and treatment.

**Patient concerns:**

A 49-year-old female patient was admitted to our hospital because of weakness and poor appetite for the past 2 weeks and lower limb edema for the past 3 days. Pathological examination of the renal biopsy specimen showed large cellular crescents and the pathology of the thyroid tissue revealed papillary thyroid carcinoma.

**Diagnoses:**

This patient was diagnosed with AAV with crescentic glomerulonephritis complicated and papillary thyroid carcinoma.

**Interventions:**

Treatment with 500 mg/day methylprednisolone for 3 days (A total of 2 times, with an interval of 2 weeks between each time)and then 40 mg/day intravenously, cyclophosphamide 1 g pulse therapy and plasma exchange, hemodialysis and resection of the left lobe of the thyroid gland. Interventions, such as anti-infection, correct anemia and electrolyte imbalance, were administered.

**Outcomes:**

After treatment, Urine output had returned to normal, the blood creatinine level had remained below 200 µmol/L, and the patient was no longer dependent on hemodialysis. Her PR3 and c-ANCA turned negative. The thyroid cancer did not recur after follow-up of 1 year.

**Lessons:**

AAV combined with papillary thyroid carcinoma is very rare, especially AAV with crescentic glomerulonephritis and papillary thyroid carcinoma. The pathogenesis between the 2 is still not clear. In addition to considering immunosuppressive agents, it is still necessary to consider the common pathogenic pathways of AAV itself or between AAV and cancers. When diagnosing AAV, attention should be paid to screening for cancer, and multi-disciplinary collaboration is required for diagnosis and treatment.

## 1. Introduction

Antineutrophil cytoplasm antibody (ANCA)-associated vasculitis (AAV) is a group of autoimmune diseases characterized by necrotizing inflammation of small and medium-sized blood vessels, including granulomatosis with polyangiitis (GPA), microscopic polyangiitis (MPA), and eosinophilic granulomatosis with polyangiitis (EGPA).^[1]^ Renal involvement can manifest as crescentic glomerulonephritis with pauci-immune complex deposition, leading to rapidly progressive renal failure. Patients with AAV exhibit a higher incidence of malignancy compared to the general population, with common cancers are skin cancer, hematological malignancies, and urinary tract cancers.^[1,2]^ However the association between AAV and thyroid cancer has not been previously reported. Here, we presented a rare case of AAV combined with crescentic glomerulonephritis and papillary thyroid carcinoma, along with a review of the literature on this unique condition.

## 2. Case presentation

### 2.1. Medical history

A 49-year-old Chinese woman who presented with weakness and poor appetite, for the past 2 weeks and lower limb edema for the past 3 days, was admitted to the local hospital. The blood test results were as follows: white blood cells 13.73 × 109/L, neutrophil percent 89%, hemoglobin 93 g/L, Urea nitrogen 28.7 µmol/L, serum creatinine 844 µmol/L, albumin 20.2 g/L, CRP 118.7 mg/L, ESR 120 mm/h. The patient was transferred to the nephrology and rheumatology department of Minzu Hospital of Guangxi Zhuang Autonomous Region in March 19, 2024 due to severe renal impairment.

### 2.2. Personal history

The patient was diagnosised as cerumen blockage and sinusitis at a local hospital due to hearing loss. There was no history of diabetes, cardiovascular or cerebrovascular disease, or other important organ diseases such as lung, orendocrine system diseases. There was no history of infectious disease, trauma, surgery, blood transfusion, or allergy. The vaccination regimen was unknown.

### 2.3. Physical examination

Body temperature, 36.8°C; pulse, 103 beats/min, respiration 20 times/min, blood pressure 112/76 mmHg. Clear con sciousness, congestion of conjunctiva in both eyes; no swollen face and eyelids; no distention of bilateral jugular veins; breath sounds in both lungs were coarse, a small amount of dry or moist rales. No bulge or depression in the precordium, normal heart border; heart rate was 103 beats/min, regular rhythm, no murmur or additional heart sounds, no pericardial friction rub. The abdomen was flat and soft, without tenderness, rebound tenderness, or muscle tension. The liver and spleen were not palpable or enlarged, and there was no tenderness at any ureteral point, including Upper ureteral point, Middle ureteral point and Lower ureteral point. The lower limbs showed mild pitting edema.

### 2.4. Laboratory results

Routine blood tests on March 19, 2024: white blood cells 12.69 × 109/L↑, neutrophil percent 89.6%↑, hemoglobin 78 g/L↓. Kidney function:serum creatinine 847 µmol/L↑, urea 33.6 mmol/L↑, uric acid 552 µmol/L. Electrolytes: potassium 5.5 mmol/L↑, sodium 130 mmol/L↓, chlorine 95 mmol/L↓, total calcium 1.71 mmol/L↓, phosphorus 1.62 mmol/L, bicarbonate 14 mmol/L. Liver function: total protein 53.3 g/L↓, albumin 22.7 g/L↓, globulin30.6 g/L, albumin/globulin ratio 0.74↓. CRP 118.8 mg/L↑, ESR 132 mm/h↑. Blood lipids: total cholesterol 4.09 mmol/L ↑, triglycerides 1.70 mmol/L↑, high-density lipoprotein cholesterol 0.72 mmol/L↓, low-density lipoprotein cholesterol 2.58 mmol/L↓. Her 24-hour urine volume was 400 mL↓, 24-hour urinary protein:700 mg/24 hours↑, Urine routine: urine protein (+), red blood cells (++), and white blood cells (++). Antinuclear antibody spectrum: antinuclear Antibody and others negative. Immune vasculitis: antineutrophil cytoplasmic antibodies type C (+), antineutrophil cytoplasmic antibodies type C titer S + 1:20, PR3 was positive, titers exceeds 400 RU/mL. Negative for antineutrophil cytoplasmic antibodies type P. Humoral immunity: immunoglobulin G 14.46 g/L, immunoglobulin A 2.28 g/L, immunoglobulin M 1.36 g/L, complement C3 0.95 g/L, complement C4 0.09 g/L↓. Thyroid function: Triiodothyronine (T3) 1.05 nmol/L↓, thyroxine (T4) 75.04 nmol/L, thyrotropin (TSH) 1.78 uIU/mL, free triiodothyronine (FT3) 1.91 pmol/L↓, free thyroxine (FT4) 13.45 pmol/L.

### 2.5. Imaging results

A renal ultrasound suggested that the kidneys were of normal size. Echocardiography: mild aortic valve regurgitation was identified. Ultrasonography of thyroid gland revealed bilateral substantial thyroid space (right lobe size 2.9 × 1.9 cm, left lobe size about 0.6 × 0.6 cm) (TI-RADS grade: Grade 3). The radiographs of her chest suggest Pneumonia with pulmonary edema, bilateral pleural effusion. A renal biopsy was performed for this patient.

### 2.6. Renal biopsy

**Figure 1.**
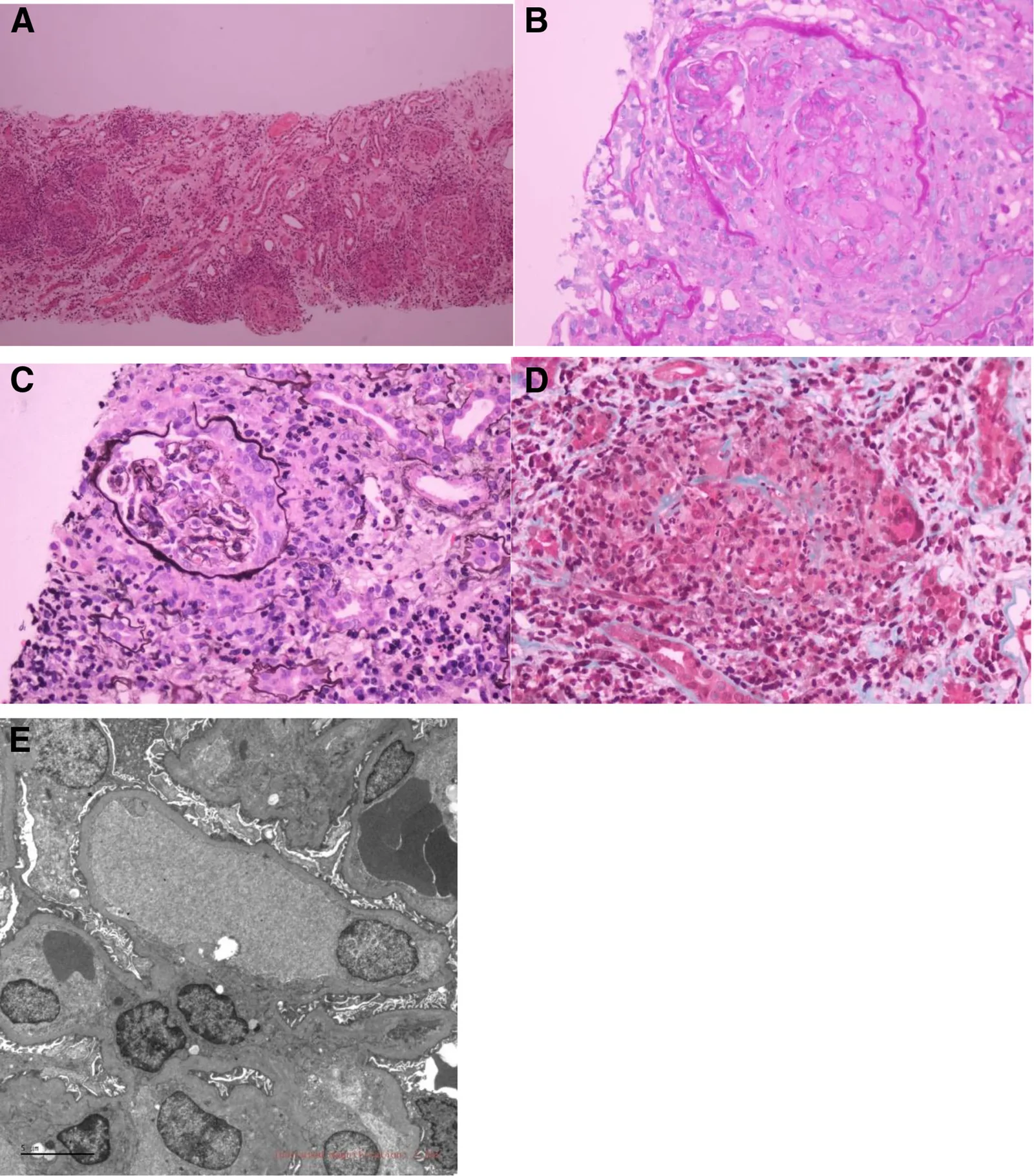
Changes of renal biopsy. (A–D) Changes of renal tissue under light microscopy; (A) hematoxylin and eosin staining (×100); (B) periodic acid-Schiff staining (×400); (C) periodic Schiff-methenamine staining (×400); (D) Masson staining (×1400). (E) Ultrastructure of renal tissue.

Light microscopy: HE, PAS, PASM, and Masson staining were performed routinely in renal biopsy tissues, including the renal cortex and medulla (Figs. 1A–D). A total of 11 glomeruli were observed, all of which were large cellular crescents (Figs. 1A–D). Vacuoles and granular degeneration were observed in tubular epithelial cells, dilatation of some renal tubular lumen, epithelial shedding, disappearance of brush edge, no obvious focal atrophy, renal interstitial edema, multi-focal inflammatory cell infiltration, no obvious fibrosis, and thickening of arteriole wall (Figs. 1A–D). The lumen was narrow and no fibrinous necrosis of the tube wall was observed (Figs. 1A–D). Electron microscopy revealed that the capillary loops were compressed, the lumens was narrowed, the basement membrane was not significantly thickened, the foot processes were partially fused, the mesangial cells and matrix show mild hyperplasia, the renal tubular epithelial cells exhibit granular degeneration, a few microvilli of the renal tubules are shed, scattered inflammatory cell infiltration is observed in the renal interstitium, and no electron-dense deposits were noted (Fig. 1E). Immunofluorescence: IgM (1+), IgG (±), IgA, C3, and C1q (−).

### 2.7. Diagnosis and treatments

The patient was diagnosed with AAV with crescentic glomerulonephritis based on medical history, symptoms, and renal biopsy. This patient was immediately administered 500 mg/day methylprednisolone for 3 days (A total of 2 times, with an interval of 2 weeks between each time) and then 40 mg/day intravenously, cyclophosphamide 1 g pulse therapy (total 2 times, with a 2-week interval between each) and plasma exchange, hemodialysis was given. Plasma exchange was performed 3 times and a total of 3700 mL of the plasma was exchanged. Her urine output had gradually returned to around 1200 to 1500 mL per day, the blood creatinine level had remained below 200 µmol/L, and the patient was no longer dependent on hemodialysis. Her PR3 and c-ANCA turned negative. She was discharged from the hospital with 25 mg/day prednisone and regularly return to the hospital for intravenous cyclophosphamide treatment. The level of steroids gradually decreased and the cumulative dose of cyclophosphamide was 5.9 g.

**Figure 2.**
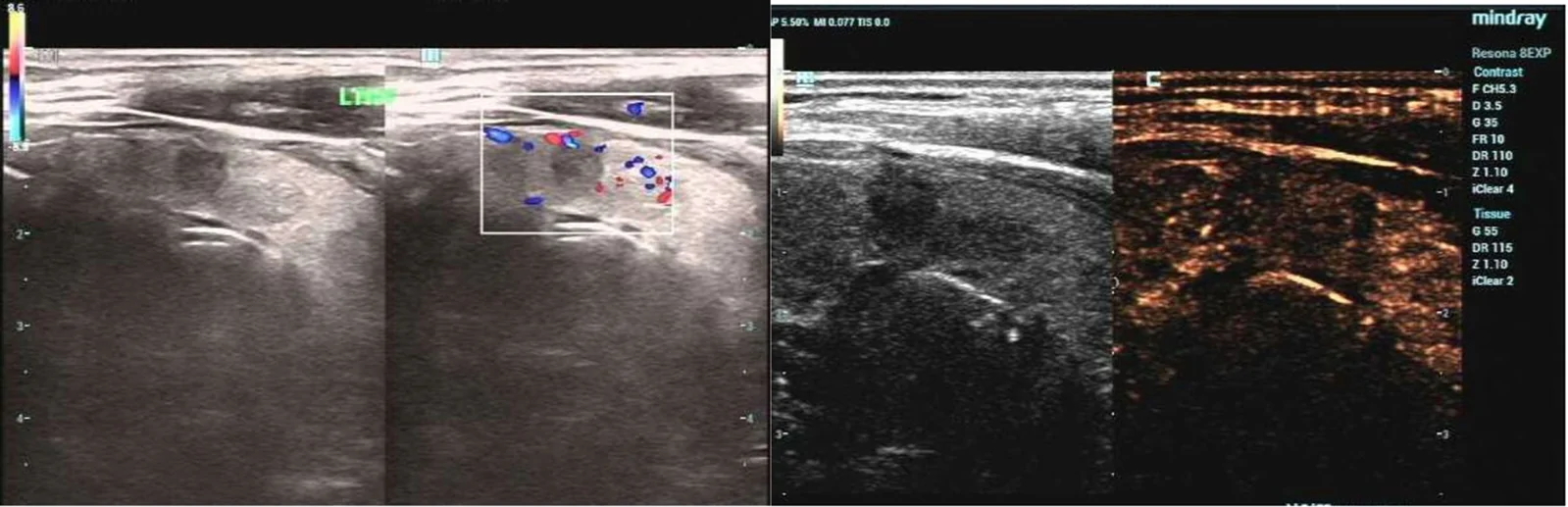
Contrast-enhanced thyroid ultrasound showed a hypoechoic mass in the left lobe of the thyroid and a mixed echo mass in the right lobe of the thyroid.

**Figure 3.**
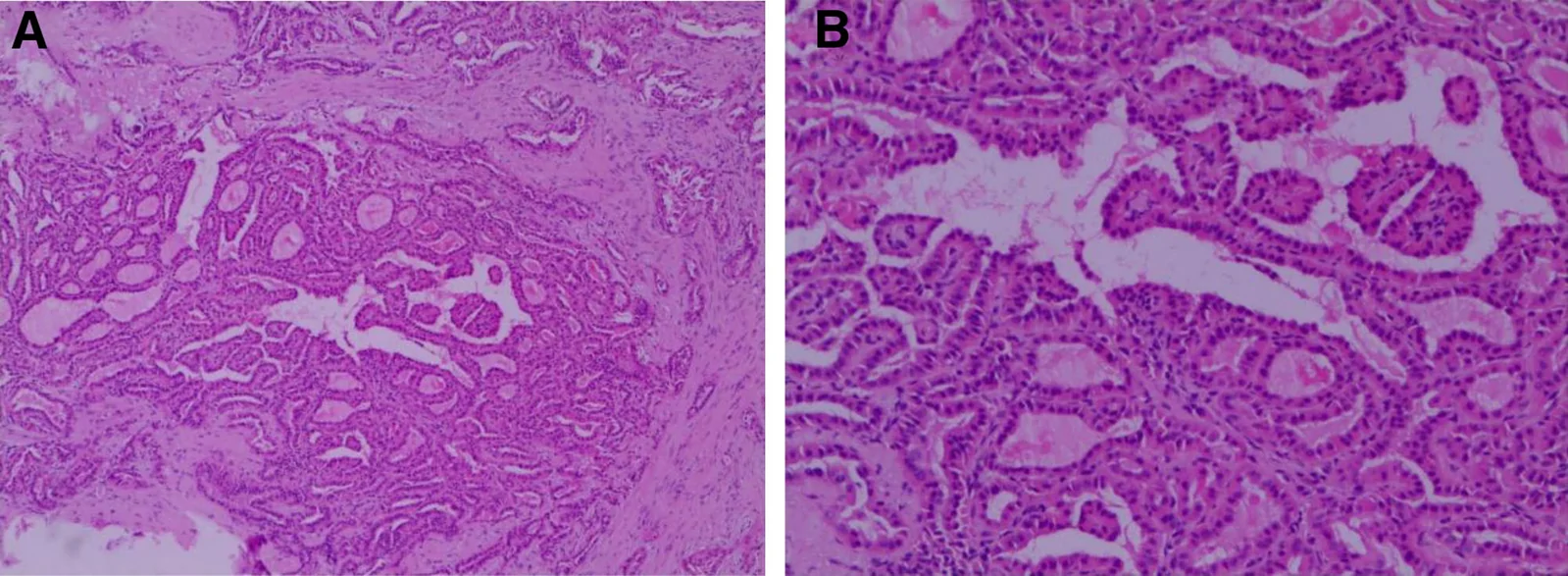
(A and B) The pathology of the thyroid tissue revealed papillary thyroid carcinoma. (A) hematoxylin and eosin staining (×4); (B) hematoxylin and eosin staining (×10).

In July 2024, the patient returned to the hospital and was treated with cyclophosphamide. At this point, the patient’s cumulative dose of cyclophosphamide was 4.3 g. At this time, contrast-enhanced thyroid ultrasound showed a hypoechoic mass in the left lobe of the thyroid (about 3.0 × 1.9 cm in size), and a mixed echo mass in the right lobe of the thyroid (about 0.6 × 0.5 cm in size) (Fig. 2). She was transferred to the Department of Thoracic, and Gland Surgery for a left thyroid mass resection, the pathology of the thyroid tissue revealed papillary thyroid carcinom (Fig. 3).

### 2.8. Follow-up after treatments

**Figure 4.**
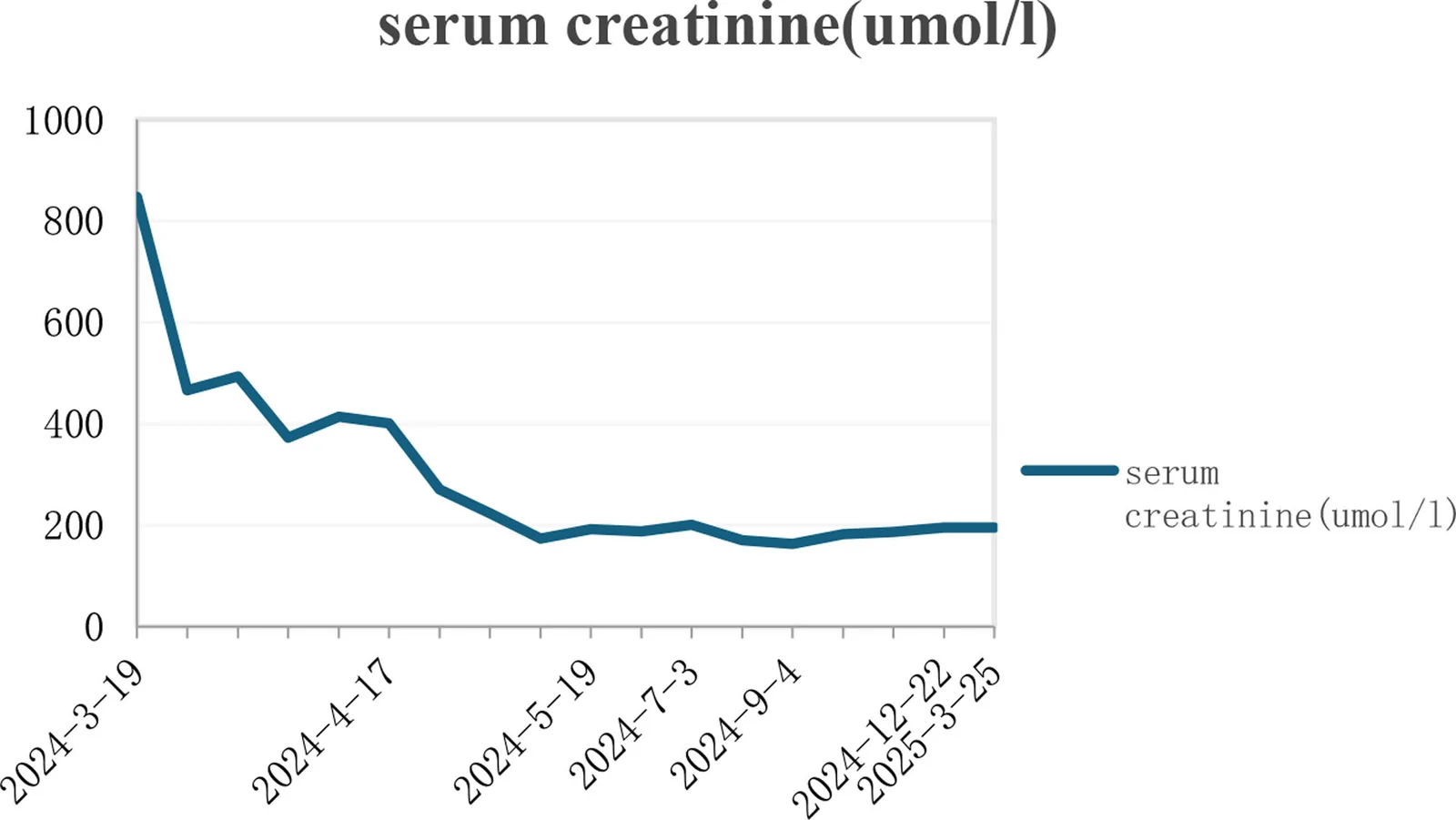
The decline in serum creatinine during treatment.

After 1 years of follow-up, presently, 10 mg once every other day oral prednisone is being provided. Her serum creatinine level has slowly decreased to 196 µmol/L, c-ANCA and PR3 is negative, 24-h urine protein level is 230 mg/24 hours and thyroid ultrasound shows postoperative changes in the thyroid gland, and no new space-occupying lesions are found. The changes in the serum creatinine level during the treatment are depicted in Figure 4.

## 3. Discussion

AAV with crescentic glomerulonephritis is a severe kidney disease. Its pathological features include the formation of crescents within glomeruli and fibrinoid necrosis, often leading to acute kidney injury or even end-stage renal disease. In this case, the patient presented with typical renal damage of AAV and was diagnosed with crescentic glomerulonephritis through renal biopsy. Additionally, the patient was also found to have papillary thyroid carcinoma, which is a rare combination that prompted us to deeply consider the association between AAV and tumors. Patients with AAV have a higher risk of developing cancers compared to the general population. The common types of cancers include skin cancer, hematological malignancies, bladder cancer, lung cancer, and prostate cancer.^[1,2]^ Papillary thyroid carcinoma is extremely rare in AAV patients, with no relevant case reports at presently. This study reports the first case of AAV with crescentic glomerulonephritis complicated and papillary thyroid carcinoma, aiming to promote early cancers screening in AAV patients. The mechanism underlying the association between AAV and cancers remains unclear.

Studies have shown that the risk of developing tumors in AAV patients is associated with immune system disorders and the use of immunosuppressive therapy, especially cyclophosphamide. Patients exposed to cyclophosphamide have a higher incidence of cancers.^[1]^ In our patient, her thyroid ultrasound indicated abnormalities before the treatment with cyclophosphamide, suggesting that immunosuppressive therapy is not the only factor leading to malignancy in patients with AAV. It may be related to the long-term inflammatory defects and immune system activation associated with AAV. AAV can induce a high level of inflammatory response, causing vascular endothelial damage, and at the same time, it may also cause gene mutations in surrounding tissues. This persistent inflammatory response has a potential carcinogenic risk.^[3]^ In this case, the patient was diagnosed crescentic glomerulonephritis secondary to AAV, suggesting severe inflammatory response. Some studies have proposed that several cytokines with potential impacts may contribute to the carcinogenesis of AAV patients. These cytokines and targeted mechanisms may also be applicable to papillary thyroid carcinoma.

AAV, as an autoimmune disease, leads to long-term immune activation, resulting in immune system damage and abnormal expression. Abnormal T cell activation is the core of the development in AAV. In AAV patients, CTLA-4 and CD25 are significantly expressed, while CD28 is lowly expressed. Meanwhile, a large amount of IFN-γ is released. The high expression of CTLA-4 and low expression of CD28 are markers of early T cell senescence and exhaustion.^[3]^ In addition to T cell exhaustion, T cells in AAV patients may also suffer damage. A study^[4]^ suggests that the induced response of T cells to mitogens in AAV patients is significantly reduced. Moreover, these T cells exhibit defective expression of CTLA-4 and high expression of IFN-γ, and there are also abnormalities in the secretion of IL-10. This evidence indicates that activated T cells are severely impaired. The damaged and exhausted T cells have a reduced ability to respond to homologous antigens, and they cannot quickly identify and eliminate tumor cells, resulting in immune escape.^[3]^

In patients with AAV, due to the molecular defect of CMTM6 (a transmembrane protein that protects PD-L1) induced by AAV, the expression of PD-L1 in monocytes is significantly downregulated, which increases the degradation of PD-L1. The expression of IL-10 is abnormally elevated in patients with AAV, promoting the production of TGF-β (a growth factor that promotes tumorigenesis), downregulating the expression of MHC in antigen-presenting cells, thereby reducing or inhibiting antigen presentation. This is considered to contribute to immunosuppression within the tumor microenvironment and promote tumor escape. In addition, IL-6 can induce IL-17 helper T cells (Th17), thereby activating neutrophils, playing an important role in the occurrence and development of AAV.^[3]^ In addition, IL-6 can induce tumor growth and metastasis through JAK/STAT, Ras/MAPK and PI3K/AKT pathways.^[5]^ In summary, AAV leads to the abnormal expression of multiple cytokines and receptors. The abnormal expression of IL-6, IL-10, and PD-L1 may be an important reason for the high risk of tumors in AAV patients.

In our patient, when she was diagnosed with AAV, thyroid ultrasound had already indicated abnormalities, suggesting that thyroid cancer may have occurred before AAV or that they coexisted. This indicates that there may be a common pathophysiological basis between AAV and tumors, namely, based on the acquired immune dysfunction state of both diseases, AAV patients have a higher inherent risk of developing tumors.

Research shows that cancer may express structures similar to neutrophil antigen epitopes (such as myeloperoxidase [MPO] or proteinase 3 [PR3]), inducing the production of ANCA and thus triggering vasculitis, for example, cases of MPO-ANCA positive vasculitis have been observed in patients with breast cancer and renal pelvis cancer.^[6]^ This molecular mimicry phenomenon suggests that tumor-associated antigens may activate the autoimmune response through cross-reaction. Cytokines (such as IL-1β, IL-18) or antigens released by tumor cells may activate neutrophils, leading to the occurrence of AAV.^[7]^ Previous studies have found that thyroid cancer is the most common cancer associated with cancer-related vasculitis. It is speculated that the abnormally produced pro-inflammatory cytokines by tumor cells, the abnormal production of antibodies and tumor neoantigens leading to abnormal immune responses, as well as the immunopathological basis such as the similarity between deposits in the blood vessel walls, tumor antigens and endothelial cell antigens are important mechanisms of tumor-induced vasculitis.^[8,9]^

In addition, genetic and epigenetic factors are also believed to be involved in increasing the risk of AAV and cancers. Studies have shown that HLA gene polymorphisms, such as HLA-DQ alleles, can increase the susceptibility to AAV and may also affect the efficiency of cancer antigen presentation.^[10]^ Abnormal DNA methylation or histone modification may also jointly contribute to the autoimmune dysfunction in AAV and the immune escape of tumor cells.^[11]^

Meanwhile, some studies have suggested that there are abnormal genes associated with thyroid cancer in some AAV patients, for example, the BRAF V600E mutation (common in papillary thyroid cancer) may interact with the signaling pathways related to ANCA production (such as NF-κB).^[12]^ In addition, p53 gene inactivation has been reported in both AAV-related vascular injury and thyroid cancer, which may reflect a common genetic background.^[13]^

## 4. Conclusion

In summary, AAV combined with papillary thyroid carcinoma is very rare, especially AAV with crescentic glomerulonephritis and papillary thyroid carcinoma. The pathogenesis between the 2 is still not clear. In addition to considering immunosuppressive agents, it is still necessary to consider the common pathogenic pathways of AAV itself or between AAV and cancers. When diagnosing AAV, attention should be paid to screening for cancer, and multi-disciplinary collaboration is required for diagnosis and treatment.

## Acknowledgments

All authors contributed to the preparation, review, and approval of the manuscript and have consented to its submission for publication. This study was supported by grants from Scientific Research Team Incubation Project (Minke Fy202110).

## Author contributions

**Data curation:** Juan Chen, Linna Lu.

**Formal analysis:** Juan Chen, Linna Lu.

**Funding acquisition:** Xin Xiang.

**Investigation:** Kun He, Diansheng Huang, Xin Xiang.

**Methodology:** Juan Chen.

**Resources:** Juan Chen, Linna Lu.

**Supervision:** Kun He, Diansheng Huang.

**Validation:** Zhandong Meng, Yuanfa Li.

**Writing – original draft:** Juan Chen.

**Writing – review & editing:** Juan Chen.
